# E3 Ubiquitin Ligase NEDD4 Promotes Influenza Virus Infection by Decreasing Levels of the Antiviral Protein IFITM3

**DOI:** 10.1371/journal.ppat.1005095

**Published:** 2015-08-11

**Authors:** Nicholas M. Chesarino, Temet M. McMichael, Jacob S. Yount

**Affiliations:** Department of Microbial Infection and Immunity, Center for Microbial Interface Biology, The Ohio State University, Columbus, Ohio, United States of America; Mount Sinai School of Medicine, UNITED STATES

## Abstract

Interferon (IFN)-induced transmembrane protein 3 (IFITM3) is a cell-intrinsic factor that limits influenza virus infections. We previously showed that IFITM3 degradation is increased by its ubiquitination, though the ubiquitin ligase responsible for this modification remained elusive. Here, we demonstrate that the E3 ubiquitin ligase NEDD4 ubiquitinates IFITM3 in cells and *in vitro*. This IFITM3 ubiquitination is dependent upon the presence of a PPxY motif within IFITM3 and the WW domain-containing region of NEDD4. In NEDD4 knockout mouse embryonic fibroblasts, we observed defective IFITM3 ubiquitination and accumulation of high levels of basal IFITM3 as compared to wild type cells. Heightened IFITM3 levels significantly protected NEDD4 knockout cells from infection by influenza A and B viruses. Similarly, knockdown of NEDD4 in human lung cells resulted in an increase in steady state IFITM3 and a decrease in influenza virus infection, demonstrating a conservation of this NEDD4-dependent IFITM3 regulatory mechanism in mouse and human cells. Consistent with the known association of NEDD4 with lysosomes, we demonstrate for the first time that steady state turnover of IFITM3 occurs through the lysosomal degradation pathway. Overall, this work identifies the enzyme NEDD4 as a new therapeutic target for the prevention of influenza virus infections, and introduces a new paradigm for up-regulating cellular levels of IFITM3 independently of IFN or infection.

## Author Summary

IFITM3 is critical for limiting the severity of influenza virus infections in humans and mice. Optimal antiviral activity of IFITM3 is achieved when it is present at high levels within cells. Our results indicate that the E3 ubiquitin ligase NEDD4 decreases baseline IFITM3 levels by ubiquitinating IFITM3 and promoting its turnover. Depleting NEDD4 from cells results in IFITM3 accumulation and greater resistance to infection by influenza viruses. Therefore, we have identified NEDD4 as a regulator of IFITM3 levels and as a novel drug target for preventing influenza virus and other IFITM3-sensitive virus infections.

## Introduction

Interferon (IFN)-induced transmembrane protein 3 (IFITM3) is a 15 kDa protein that restricts cellular infection by influenza virus [1,2,3]. IFITM3 is active against all strains of influenza virus that have been tested to date, regardless of serotype or species of origin [1,3,4,5,6], and it similarly inhibits many other medically important viruses such as HIV, SARS coronavirus, and Ebola virus [1,5,7,8,9]. Confirming its importance *in vivo*, IFITM3 knockout mice succumb to sublethal doses of influenza virus [10,11]. Likewise, IFITM3 is the only known protein for which a genetic polymorphism present in a significant percentage of the human population is associated with severe influenza virus infections [10,12,13,14]. In the cell, IFITM3 localizes to endosomes and lysosomes [15,16,17], and traps endocytosed virus particles within these degradative compartments by impeding the formation of the virus fusion pore [16,18,19]. Yet, even with this potent mechanism by which IFITM3 limits infections, influenza virus remains a significant health concern [20,21]. This may be explained by the fact that IFITM3 is present at low levels within most cells at steady state and is induced by IFNs only after infection has already been established [3,11,22]. The inability to up-regulate IFITM3 levels independently of infection or IFNs is a challenge preventing the field from harnessing the activity of IFITM3 for infection prevention.

We previously showed that ubiquitination increases the rate of IFITM3 turnover within the cell [15]. A non-ubiquitinated lysine-to-alanine mutant of IFITM3 possessed enhanced antiviral activity and a longer half-life as compared to WT IFITM3 [15]. These findings indicated that inhibition of IFITM3 ubiquitination could augment the activity and/or levels of endogenous IFITM3, thus offering a strategy for exploiting IFITM3 therapeutically or prophylactically against viral infections. The identification of the E3 ubiquitin ligase(s) capable of modifying IFITM3 among the more than 600 annotated E3 ligases in the human genome will be an important step toward validating this antiviral strategy.

**Fig 1:**
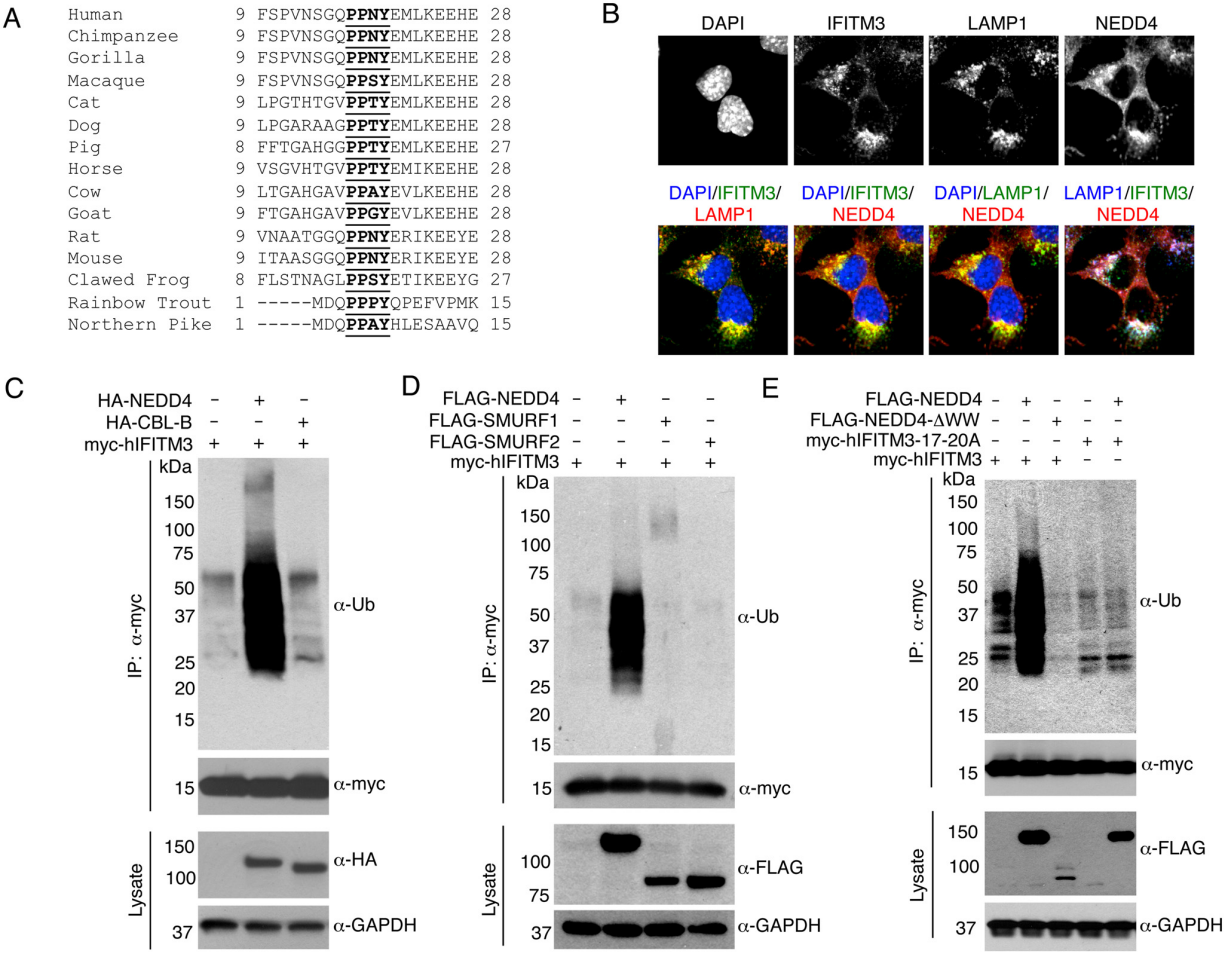
IFITM3 is ubiquitinated by NEDD4. A) Alignment of IFITM3 N-terminal amino acids from various species. Bold and underlined text highlights the conserved PPxY motif. B) Mouse embryonic fibroblasts (MEFs) were stimulated overnight with IFN-α (160 units/mL) to ensure production of IFITM3, and imaged by fluorescent confocal microscopy with staining for endogenous IFITM3, NEDD4, LAMP1, and nuclei (DAPI). Images were taken with a 60x objective and 2.5x zoom. Pseudocolored merged images in different staining combinations are shown. C-E), HEK293T cells were co-transfected with plasmids expressing IFITM3 and epitope tagged ubiquitin ligases, NEDD4, CLB-B, SMURF1 and SMURF2, as indicated. Cell lysates were immunoprecipited with anti-myc resin, and examined by Western blotting with anti-myc and anti-ubiquitin (Ub) antibodies. Western blots of cell lysates with anti-HA (C) or anti-FLAG (D,E) antibodies were performed to confirm expression of the ubiquitin ligases. Anti-GAPDH Western blotting was performed to confirm comparable protein loading.

Through our work studying tyrosine phosphorylation of IFITM3, we discovered that phosphorylation at tyrosine 20 (Y20) inhibited IFITM3 ubiquitination [23]. This led us to posit that phosphorylation of Y20 may block an E3 ubiquitin ligase recognition signal. Indeed, Y20 is part of a highly conserved PPxY motif (where P = proline, x = any amino acid, and Y = tyrosine, Fig 1A)[24]. PPxY motifs are commonly recognized by WW (characterized by two tryptophan residues spaced approximately 20 amino acids apart) domains of NEDD4-family E3 ubiquitin ligases, of which there are nine family members [25]. We chose to focus first on NEDD4, the prototypical member of this family, for several reasons: 1) NEDD4 and IFITM3 both have ubiquitous expression patterns while several other NEDD4-family members are tissue-specific (BioGPS.org [26]), 2) Like IFITM3, many of the known NEDD4 substrates are membrane proteins and are associated with endosomal and lysosomal pathways [25,27], 3) IFITM3 and NEDD4 are both S-palmitoylated, suggesting that they may localize to similar membrane subdomains [2,28], and 4) NEDD4 is reported to be inhibited by ISG15 [29,30,31], an IFN-inducible protein, thus providing an intriguing model whereby IFN might induce IFITM3 expression while also inhibiting its ubiquitination. Herein, we provide results demonstrating the ability of NEDD4 to ubiquitinate IFITM3 and identify a unique role for NEDD4 in decreasing steady state IFITM3 abundance, leading to increased cellular susceptibility to influenza virus infection.

## Results

### NEDD4 co-localizes with IFITM3 at lysosomes

To explore the possibility that NEDD4 ubiquitinates IFITM3, we first examined whether IFITM3 and NEDD4 are in proximity to one another within cells. We stimulated mouse embryonic fibroblasts (MEFs) with IFN-α to induce abundant expression of IFITM3. By performing immunofluorescence microscopy imaging of endogenous IFITM3 and NEDD4, we detected co-localization of these two proteins (Fig 1B). Further co-localization of these proteins with endogenous LAMP1, a lysosomal marker, indicates that NEDD4 and IFITM3 may interact at lysosomes (Fig 1B).

### NEDD4 overexpression increases IFITM3 ubiquitination

We next examined the effect of overexpressing HA-tagged human NEDD4 (HA-NEDD4) on IFITM3 ubiquitination. We observed a significant increase in myc-tagged human IFITM3 (myc-hIFITM3) ubiquitination when HA-NEDD4 was expressed as compared to the transfection control (Fig 1C). On the contrary, no increase in IFITM3 ubiquitination was seen upon overexpression of HA-tagged human CBL-B, another E3 ubiquitin ligase that has been reported to interact with NEDD4 [32], and that is associated with regulation of immune responses [33] (Fig 1C). Additionally, we examined the effect of overexpressing FLAG-tagged human NEDD4 (FLAG-NEDD4) on IFITM3 ubiquitination in comparison to FLAG-tagged human SMURF1 and SMURF2 (FLAG-SMURF1 and FLAG-SMURF2), both of which are members of the NEDD4-family of ubiquitin ligases. FLAG-NEDD4 caused an increase in IFITM3 ubiquitination while FLAG-SMURF1 and FLAG-SMURF2 were unable to robustly modify IFITM3 (Fig 1D). These results demonstrate that NEDD4 possesses a degree of specificity for IFITM3 that is lacking for CBL-B and the NEDD4-family members, SMURF1 and SMURF2.

### The IFITM3 PPxY motif is required for ubiquitination by NEDD4

As previously mentioned, NEDD4-family ubiquitin ligases possess two to four characteristic WW domains that interact with proline-rich motifs, including PPxY motifs, on substrate proteins [34]. NEDD4 has four WW domains and IFITM3 contains a highly conserved PPxY motif within its N-terminus (Fig 1A). To test whether these domains are required for IFITM3 ubiquitination, we generated an IFITM3 mutant in which each residue of the PPxY motif (17-PPNY-20 in IFITM3) was mutated to alanine (designated 17-20A) and utilized a FLAG-NEDD4 mutant in which its four WW domains were deleted (designated ΔWW). Upon co-overexpression of FLAG-NEDD4 with myc-hIFITM3, ubiquitination of IFITM3 was increased as expected, while the ΔWW mutant was unable to increase IFITM3 ubiquitination (Fig 1E). In fact, FLAG-NEDD4-ΔWW partially decreased steady state IFITM3 ubiquitination, perhaps indicating a dominant negative effect (Fig 1E). Moreover, the 17-20A mutant of IFITM3 showed less ubiquitination than WT IFITM3 and was unaffected by overexpression of NEDD4 (Fig 1E).

**Fig 2:**
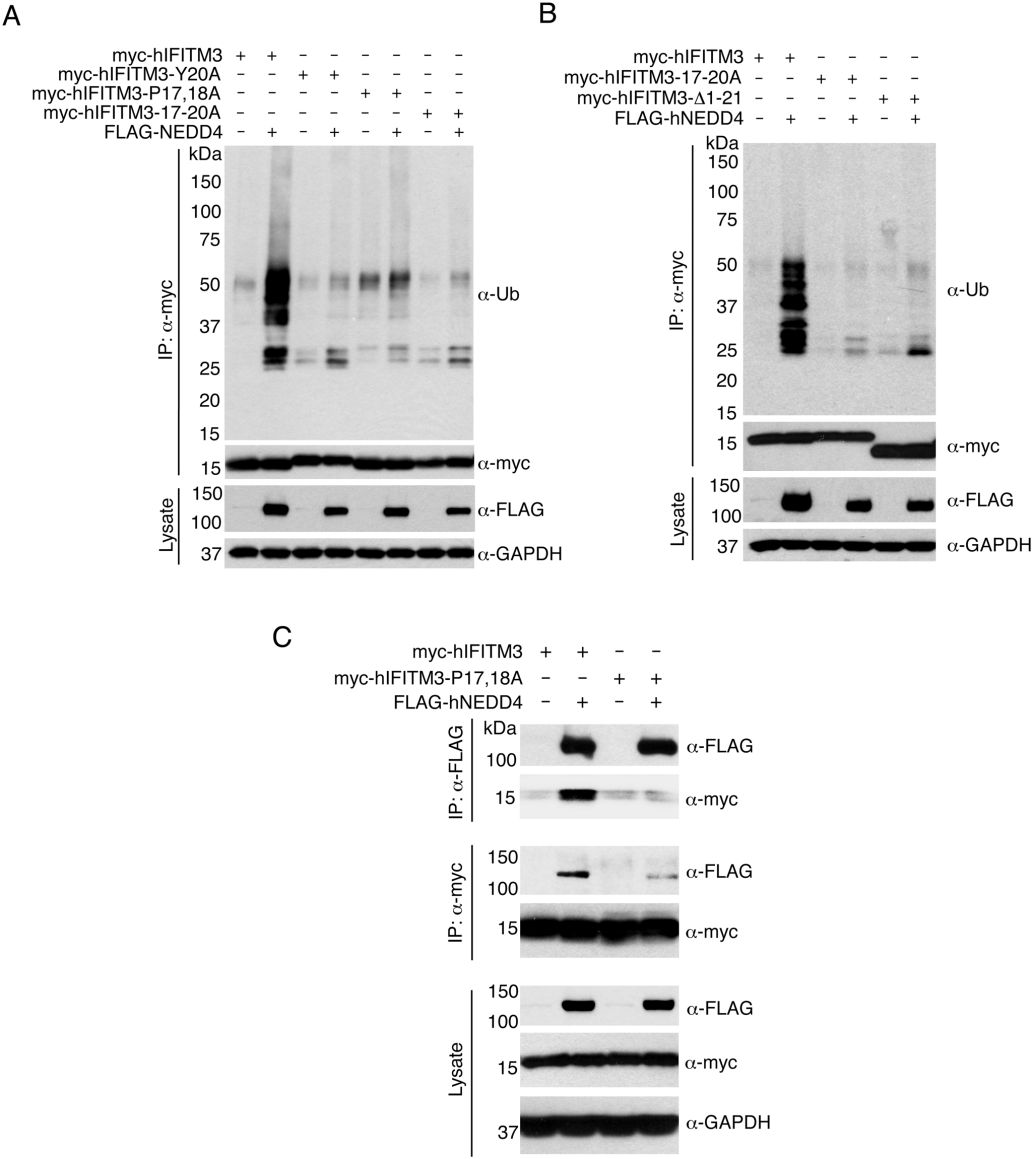
The IFITM3 PPxY motif is required for ubiquitination by NEDD4. A-C) HEK293T cells were co-transfected with plasmids expressing myc-hIFITM3 or FLAG-NEDD4 as indicated. A-B) Cell lysates were immunoprecipitated with anti-myc resin, and examined by Western blotting with anti-myc and anti-ubiquitin (Ub). Western blotting of cell lysate with anti-FLAG antibodies was performed to confirm expression of NEDD4. Western blotting with anti-GAPDH antibodies was performed to confirm comparable protein loading. C) Cell lysates were immunoprecipitated with anti-myc or anti-FLAG resin, and co-immunoprecipitation was examined by Western blotting with both anti-myc and anti-FLAG antibodies for each immunoprecipitate. Western blots of cell lysates with anti-myc and anti-FLAG antibodies were performed to confirm expression of IFITM3 and NEDD4, respectively. Anti-GAPDH Western blotting was performed to confirm comparable protein loading.

The IFITM3 PPxY motif shares its tyrosine with an overlapping YxxΦ motif known to be involved in the trafficking of IFITM3 from the plasma membrane to endosomes [23,35,36]. Thus, in order to be certain that the results we observed for the 17-20A mutant of IFITM3 was not because of interference with the YxxΦ motif, we tested additional PPxY mutants in which the two prolines were mutated to alanine (myc-hIFITM3-P17,18A) or in which the tyrosine was mutated to alanine (myc-hIFITM3-Y20A). Upon co-overexpression of FLAG-NEDD4, the ubiquitination of both of these mutants was only minimally increased as compared to the robust increase in ubiquitination of WT IFITM3 (Fig 2A). Interestingly, a truncated form of IFITM3 missing its first 21 amino acids, including the PPxY motif, is prevalent in certain human populations. This variant is associated with severe influenza virus infections [10,13,14] and more rapid progression of HIV-related disease [37]. A myc-hIFITM3 construct lacking these first 21 amino acids (Δ1–21) was, as expected, largely unaffected in terms of ubiquitination by overexpression of FLAG-NEDD4 (Fig 2B), identifying a potentially important difference between the truncated and full-length IFITM3 proteins.

Next, since NEDD4 has been shown to physically interact with the PPxY motifs of its substrate proteins [25], we examined whether or not NEDD4 and IFITM3 co-immunoprecipitate with one another. We found that myc-hIFITM3 and FLAG-NEDD4 indeed co-immunoprecipitated with one another (Fig 2C), suggesting a physical interaction. Importantly, this interaction was greatly diminished between FLAG-NEDD4 and the P17,18A mutant of IFITM3 (Fig 2C). In sum, these results indicate that the IFITM3 PPxY motif is required for a strong interaction with and ubiquitination by NEDD4.

### NEDD4-mediated IFITM3 ubiquitination is dependent upon NEDD4 catalytic activity

**Fig 3:**
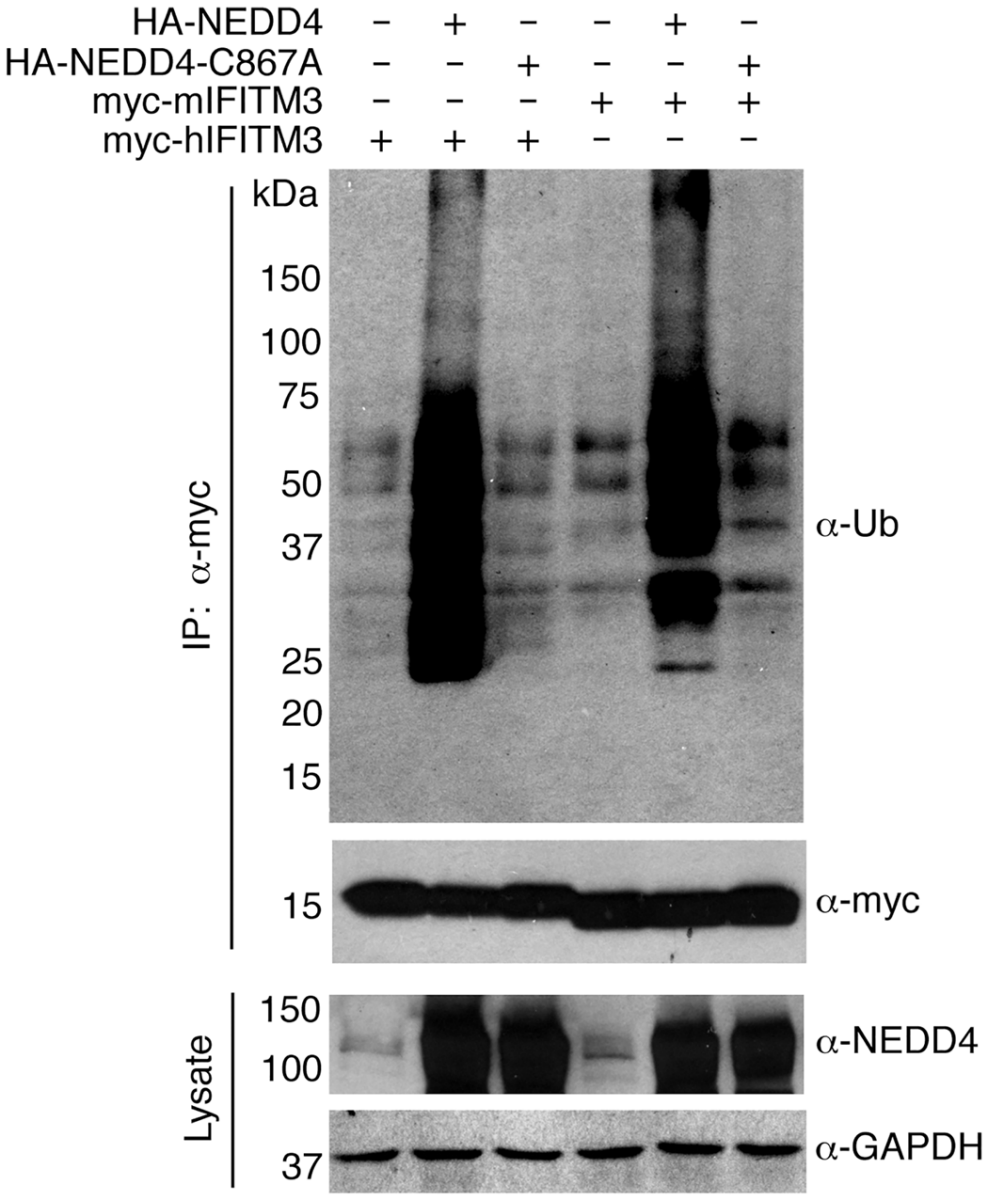
NEDD4 catalytic activity is required for IFITM3 ubiquitination. HEK293T cells were transfected with the indicated mouse or human IFITM3 constructs and were co-transfected with plasmids expressing HA-NEDD4 or a catalytically inactive HA-NEDD4-C867A mutant. IFITM3 was immunoprecipitated with anti-myc resin and subjected to anti-myc and anti-ubiquitin (Ub) Western blotting. Cell lysates were probed with anti-NEDD4 antibodies to confirm expression of NEDD4 constructs. Anti-GAPDH staining served as a protein loading control.

To determine whether a non-enzymatic activity of NEDD4 might be mediating its effect on IFITM3 ubiquitination, we tested a catalytically inactive NEDD4 point mutant. We found that this mutant was unable to increase IFITM3 ubiquitination, establishing that catalytic activity of NEDD4 is indeed required for its ability to increase IFITM3 ubiquitination (Fig 3). Since murine (m)IFITM3 also possesses a PPxY motif (Fig 1A), we tested the ability of NEDD4 to affect mIFITM3 modification. Like myc-hIFITM3, we observed an increase in myc-mIFITM3 ubiquitination when HA-NEDD4 was co-overexpressed and observed no effect of the catalytic mutant (Fig 3), suggesting a possible evolutionary conservation of NEDD4 modification of IFITM3 in mice and humans. These data further implicate NEDD4 as an E3 ubiquitin ligase capable of enzymatically modifying mouse and human IFITM3.

### NEDD4 can modify IFITM3 *in vitro*

**Fig 4:**
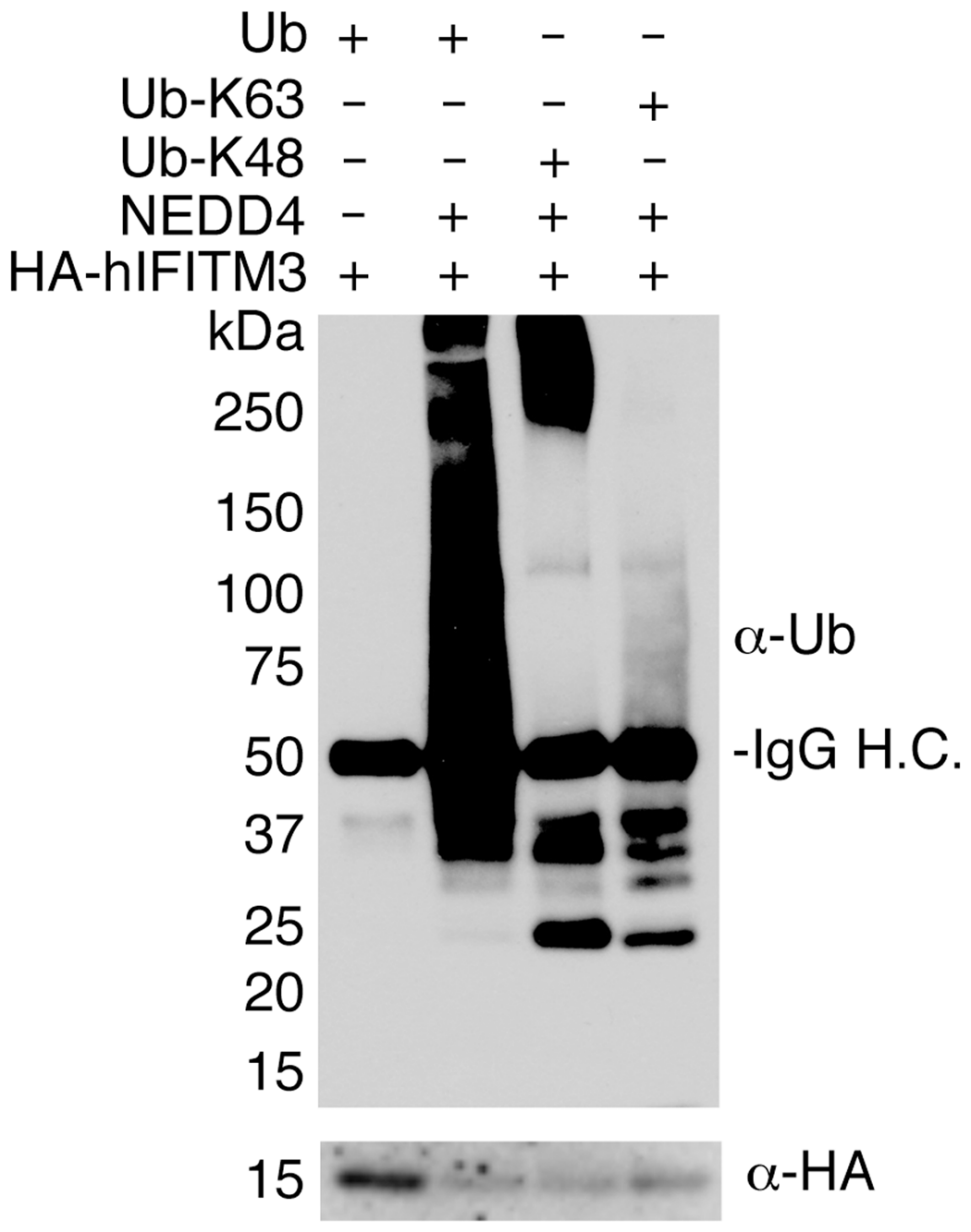
NEDD4 ubiquitinates IFITM3 in vitro. HA-hIFITM3 was added to reactions containing NEDD4-compatible E1 and E2 ubiquitin ligases, ubiquitin (WT or mutants in which only K48 or K63 were not mutated), and reaction buffer containing ATP in the presence or absence of NEDD4. The reaction was allowed to proceed for 1 h at 37°C, and IFITM3 was re-immunoprecipitated and subjected to Western blotting with anti-ubiquitin (Ub) and anti-HA antibodies. IgG H.C. indicates detection of the heavy chain of the immunoglobulin used for immunoprecipitation.

While NEDD4 overexpression experiments suggest that NEDD4 directly ubiquitinates IFITM3 (Figs 1C, 1D and 1E, 2A and 2B and 3), this effect could be indirect. We therefore tested the ability of purified NEDD4 to ubiquitinate immunoprecipitated IFITM3 *in vitro* in order to confirm that NEDD4 can directly modify IFITM3. HA-hIFITM3 was incubated with purified NEDD4, enzymatic cofactors, and ubiquitin. We then re-immunoprecipitated IFITM3 and subjected it to anti-ubiquitin western blotting. Our results show that NEDD4 is capable of robustly ubiquitinating IFITM3 *in vitro* (Fig 4). Additionally, we employed ubiquitin mutants that could only be added via lysine 48 (K48) or lysine 63 (K63) linkages in order to examine whether NEDD4 preferentially utilizes one of these polyubiquitination linkages for modifying IFITM3. While both K48 and K63 linkages could be added to IFITM3 by NEDD4, we observed a preference for the K48 linkage in long polyubiquitin chains, which is traditionally associated with protein degradation (Fig 4). These results are consistent with our past results using linkage-specific anti-ubiquitin antibodies, which demonstrated that while both K48 and K63 ubiquitin linkages could be detected on IFITM3, K48 linkages are more prevalent [15]. These data are also consistent with our previous results indicating that ubiquitination of IFITM3 promotes its turnover [15].

### NEDD4 knockout decreases IFITM3 ubiquitination and increases resistance to viral infection

**Fig 5:**
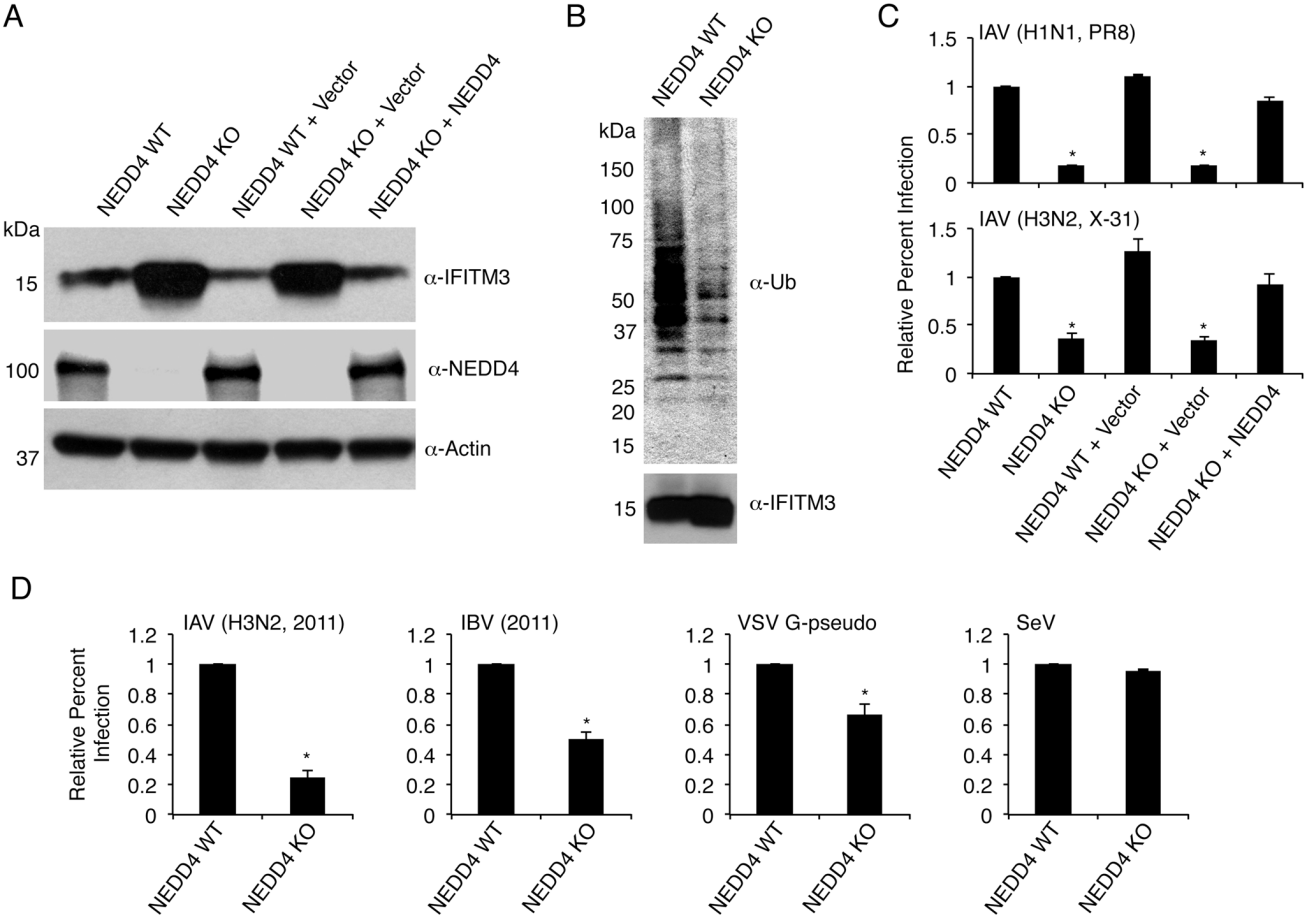
NEDD4 knockout decreases IFITM3 ubiquitination and protects cells from virus infection. A) Cell lysates from NEDD4 WT and KO MEFs were subjected to anti-IFITM3, anti-NEDD4, and anti-actin immunoblotting to evaluate NEDD4 levels in each cell line and the effect of NEDD4 on endogenous IFITM3 levels. Retroviral reconstitution of the indicated cells with an empty retrovirus control or retrovirus expressing NEDD4 is denoted by + Vector and + NEDD4, respectively. B) 2 mg of protein from NEDD4 WT and KO cell lysates were immunoprecipitated for endogenous IFITM3 and examined by Western blotting with anti-ubiquitin and anti-IFITM3 antibodies. C) The indicated cell lines were infected for 24 h with influenza A virus (IAV) PR8 and X-31 strains at an MOI of 5. Cells were then fixed and stained with anti-influenza virus NP to measure the percentage of cells infected using flow cytometry. D) NEDD4 WT and KO MEFs were infected with influenza virus 2011 isolates (IAV or influenza B virus (IBV)) or Sendai virus (SeV) at an MOI of 5 for 24 h, or were infected with VSV G-pseudotyped retrovirus (VSV G-pseudo) for 48 h. Cells were then fixed and stained with anti-influenza NP in the case of influenza virus infections or were examined for GFP positivity in the case of VSV G-pseudo or SeV to measure the percentage of cells infected using flow cytometry. C,D) Non-infected samples were used as a baseline for gating of infected cells. Results shown are representative of at least three independent experiments, each performed with triplicate samples. The average percent infection of WT NEDD4 cells was set to 1 for the calculation of relative percent infection. Error bars represent standard deviation of triplicate samples. * Indicates a p-value less than 0.001 calculated by Student’s t-test in comparison to values for NEDD4 WT cells.

In order to examine the effects of NEDD4 on endogenous IFITM3, we examined NEDD4 WT and knockout (KO) mouse embryonic fibroblasts (MEFs)[38]. We also utilized KO MEFs reconstituted with NEDD4 via retroviral transduction. Remarkably, Western blotting of lysates from NEDD4 KO cells showed an increase in steady state IFITM3 levels as compared to WT cells, while NEDD4 reconstitution decreased IFITM3 to WT levels (Fig 5A). To examine the requirement for NEDD4 in ubiquitinating IFITM3, we immunoprecipitated IFITM3 from large quantities of lysate from both WT and KO cells, expecting that the immunoprecipitation reagents would be saturated, thus providing us with comparable amounts of IFITM3 for examination of ubiquitination. Indeed, IFITM3 from NEDD4 KO cells was ubiquitinated much less than IFITM3 from WT cells (Fig 5B). These results demonstrate that NEDD4 is required for proper steady state ubiquitination of IFITM3, and that the absence of NEDD4 results in cellular accumulation of unmodified IFITM3.

Given the increase in baseline IFITM3 levels, we predicted that NEDD4 KO cells would be more resistant to influenza virus infection. We observed that NEDD4 KO MEFs were in fact significantly less susceptible to infections with influenza A virus (IAV) subtypes H1N1 and H3N2 (PR8 and X-31 strains, respectively) compared to WT control cells (Fig 5C). The decreased susceptibility of KO cells was returned to WT levels of infection upon NEDD4 reconstitution (Fig 5C). We also verified that the enhanced resistance of NEDD4 KO cells to influenza virus infection included resistance to recently circulating strains. NEDD4 KO cells were significantly less susceptible than WT cells to infection by both influenza B virus (IBV) and IAV H3N2 strains isolated in 2011 (Fig 5D). We also examined retrovirus pseudotyped with the vesicular stomatitis virus (VSV) G protein, which is also reported to be inhibited by IFITM3 [3,35,36,39,40]. As expected, the percent of NEDD4 KO cells infected with VSV G-pseudotyped virus was significantly less than WT cells (Fig 5D). Sendai virus (SeV), a parainfluenza virus that primarily fuses at the cell surface [41] and is thus only minimally affected by IFITM3 [4], was also tested. Unlike IAV, IBV, and VSV G-pseudotyped retrovirus, SeV was not appreciably affected by NEDD4 KO (Fig 5D). Thus, the pattern of virus restriction we observed is consistent with protection of NEDD4 KO cells by IFITM3.

**Fig 6:**
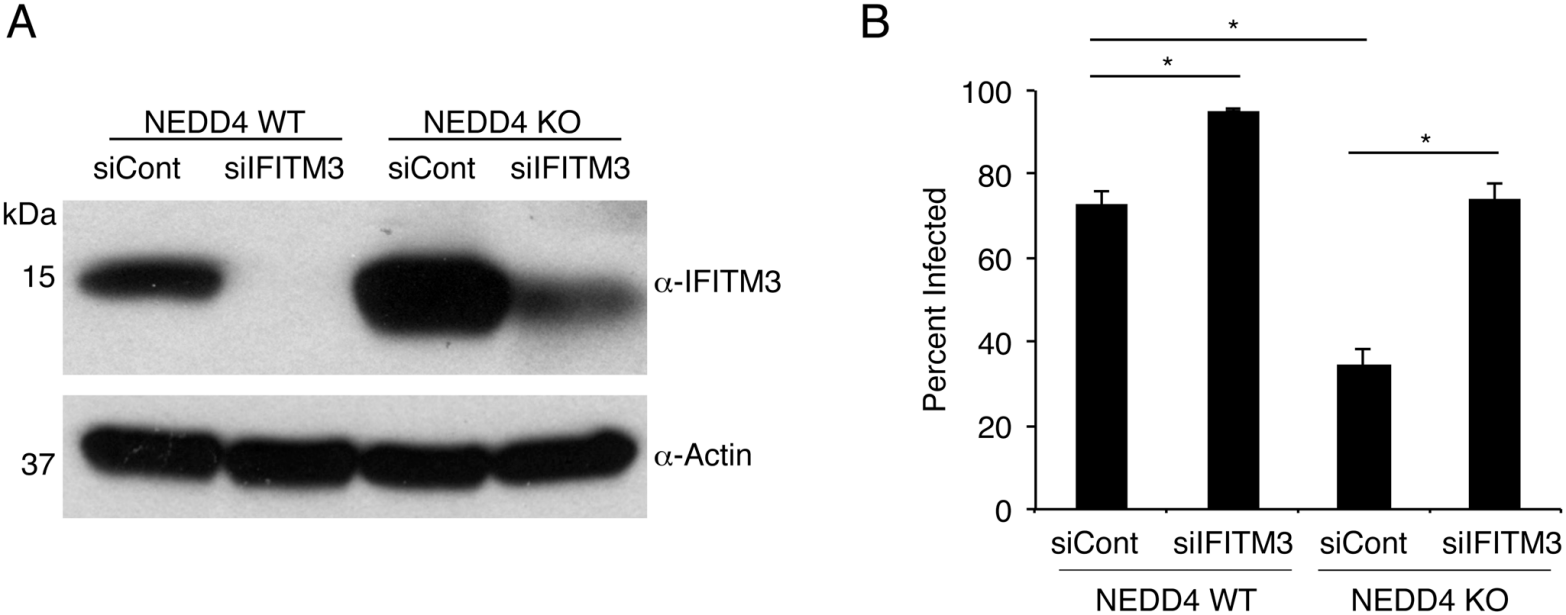
NEDD4 regulates cellular susceptibility to influenza virus infection by controlling IFITM3 levels. A,B) NEDD4 WT and KO MEFs were transfected for 24 h with control siRNA (siCont) or siRNA targeting IFITM3 (siIFITM3). A) Cells were collected just prior to infection for confirmation of IFITM3 knockdown by anti-IFITM3 Western blotting, with anti-actin blotting serving as a protein loading control. B) Following siRNA treatment, cells were infected with influenza virus strain PR8 at an MOI of 10 for 24 h. Cells were then fixed and stained with anti-influenza virus NP to measure the percentage of cells infected using flow cytometry. Results shown are representative of three independent experiments, each with samples run in triplicate. Error bars represent standard deviation. * Indicates p-value less than 0.001 calculated with Student’s t-test for comparison of samples denoted by horizontal lines.

To confirm that the increased resistance of NEDD4 KO cells to influenza virus infection was due to increased levels of basal IFITM3, we knocked down IFITM3 in NEDD4 WT and KO cells for 24 hours prior to infection. Knockdown was verified through Western blotting of cell lysates prepared at the time of infection (Fig 6A). Importantly, knockdown of IFITM3 in both NEDD4 WT and KO MEFs resulted in an increase in influenza virus susceptibility, and largely eliminated the resistance of NEDD4 KO cells to infection (Fig 6B). Overall, these experiments demonstrate that NEDD4 promotes cellular susceptibility to influenza virus infection by decreasing levels of IFITM3.

### NEDD4 knockdown in human lung cells increases IFITM3 levels and resistance to influenza virus infection

**Fig 7:**
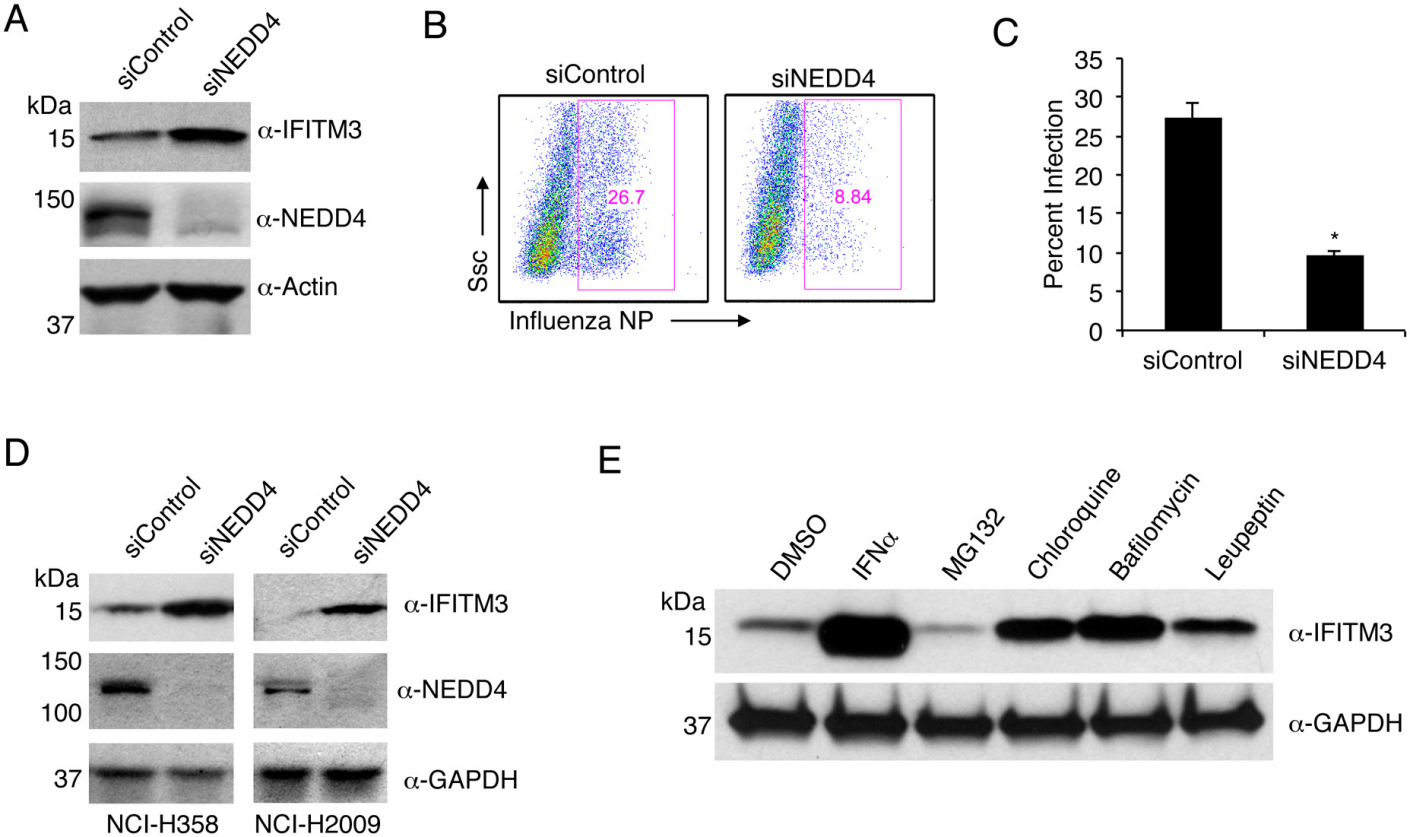
NEDD4 knockdown in human lung cells increases IFITM3 levels and resistance to influenza virus infection. A-C) A549 cells were transfected for 48 h with control siRNA (siControl) or siRNA targeting human NEDD4 (siNEDD4). A) Cells were collected just prior to infection for confirmation of NEDD4 knockdown by anti-NEDD4 Western blotting, with anti-actin blotting serving as a loading control. Anti-IFITM3 blotting demonstrates an increase in IFITM3 upon NEDD4 knockdown. B,C) Following siRNA treatment, cells were infected with influenza virus strain PR8 at an MOI of 2.5 for 6 h. Cells were then fixed and stained with anti-influenza virus NP to measure the percentage of cells infected using flow cytometry. Results shown are representative of three independent experiments, with samples run in triplicate. Error bars represent standard deviation of triplicate samples. * Indicates a p-value less than 0.0001 calculated with Student’s t-test. D) NCI-H358 and NCI-H2009 cells were transfected for 48 h with siControl or siNEDD4. Cell lysates were subjected to immunoblotting with anti-NEDD4 to confirm NEDD4 knockdown, anti-IFITM3 to demonstrate increase in endogenous IFITM3 upon NEDD4 knockdown, and anti-GAPDH as a loading control. E) A549 cells were treated with equal volumes Dimethyl Sulfoxide (DMSO) as a control, MG132 (10 μM), Chloroquine (10 μM), Bafilomycin A1 (1 μM), or Leupeptin (100 μM) for 24 h. Cells were also treated with IFN-α (100 units/mL) for comparison. Cell lysates were subjected to anti-IFITM3 immunoblotting to evaluate endogenous IFITM3 levels with each treatment. Western blotting with anti-GAPDH served as a loading control.

To extend our results to more relevant human lung cells, we utilized the A549 human alveolar epithelial cell line to study the role of NEDD4 in the regulation of steady state IFITM3 levels. Knockdown of NEDD4 with siRNA in A549 cells led to a significant increase in endogenous IFITM3 compared to non-targeting control siRNA (Fig 7A). As expected, NEDD4 knockdown led to a significantly greater resistance to IAV infection (Fig 7B and 7C). Importantly, we found that the relationship between NEDD4 knockdown and increased IFITM3 levels was preserved in two additional human lung cell lines (Fig 7D). Taken together with experiments presented in Figs 3, 5 and 6, these data confirm an evolutionary conservation between mice and humans in the regulation of cellular IFITM3 levels by NEDD4. This work also identifies NEDD4 as a novel target in human cells for improving resistance to influenza virus infection independently of IFNs or adaptive immunity.

### IFITM3 is turned over by the lysosomal degradation pathway

The degradative pathway involved in the turnover of steady state IFITM3 has not been previously investigated. Since NEDD4 is known to associate with the endosomal and lysosomal system and to target several of its substrates for lysosomal degradation [25], our results identifying NEDD4 as the primary ubiquitin ligase for IFITM3 would suggest that IFITM3 is degraded in lysosomes. To test this hypothesis, we utilized chloroquine and bafilomycin, which inhibit endosomal and lysosomal acidification and thus the activation of pH-dependent lysosomal proteases. We observed that treatment of A549 lung cells with these two inhibitors caused an accumulation of IFITM3 (Fig 7E). Similarly, treatment with leupeptin, an inhibitor of specific lysosomal proteases resulted in a similar increase in IFITM3 levels (Fig 7E). This is in contrast to the treatment of cells with the proteasomal inhibitor MG132, which consistently caused a modest decrease in IFITM3 levels, perhaps due to up-regulation of lysosomal degradation pathways when proteasome activity is inhibited. Overall, these experiments demonstrate that, consistent with the co-localization of IFITM3 and NEDD4 at lysosomes (Fig 1B), and the ubiquitination of IFITM3 by NEDD4 (Figs 1C, 1D and 1E, 2,3,4 and 5B), IFITM3 is turned over by the lysosomal degradation pathway.

## Discussion

Our previous work established that ubiquitination promotes the turnover of IFITM3 [15]. Thus, identification of the IFITM3 ubiquitin ligase would provide a potential target for increasing IFITM3 abundance and resistance to virus infections. In our previous work studying regulation of IFITM3 endocytosis by phosphorylation, we made the serendipitous discovery that the amino acid Y20 within IFITM3 is involved in regulating IFITM3 ubiquitination [23], which led us to identify the involvement of the IFITM3 PPxY motif in its ubiquitination by NEDD4 (Figs 1E and 2). NEDD4 knockdown or knockout in human or mouse cells, respectively, resulted in substantially greater levels of steady-state IFITM3 (Figs 5A, 6A and 7A and 7D). This accumulation of unmodified IFITM3 is consistent with the observed decrease in IFITM3 ubiquitination in NEDD4 KO cells (Fig 5B).

An additional intriguing aspect of our finding that IFITM3 steady state levels are regulated by NEDD4 is the previously described role of the IFN effector ISG15 in inhibiting NEDD4 [29,30]. ISG15 is a ubiquitin-like protein that specifically binds to NEDD4, blocking its productive interaction with Ubiquitin-E2 ligase complexes [29,30]. The importance of this pathway was highlighted by two independent studies demonstrating that ISG15 blocks NEDD4-mediated monoubiquitination of the VP40 matrix protein of Ebola virus, thereby inhibiting the budding of Ebola virus-like particles [29,30]. Importantly, several studies have implicated ISG15 as a critical antiviral effector against IAV and IBV [42,43,44]. Two studies have demonstrated conjugation of ISG15 onto the IAV NS1 protein by the E3 ligase HERC5, and found that ISGylation of IAV NS1 antagonizes virus replication [44,45]. Interestingly, IBV NS1 specifically blocks human ISG15 conjugation by preventing ISG15 interaction with the ISG15 activating enzyme UbE1L, effectively counteracting its antiviral effect [43,46,47,48]. We posit that high levels of IFITM3 attained after IFN stimulation result from both IFITM3 gene induction, as well as increased IFITM3 protein stability as a result of ISG15 inhibition of NEDD4. We are currently investigating this exciting potential synergistic link between ISG15 and IFITM3.

Our work demonstrates that NEDD4 is required for proper basal ubiquitination of IFITM3 (Fig 5B). However, our results would also suggest that additional ubiquitin ligases are also able to modify IFITM3, particularly when IFITM3 is present at high levels. This is supported by detection of partial ubiquitination of our various IFITM3-PPxY mutants (Figs 1E and 2A and 2B) and by detection of modest IFITM3 ubiquitination in NEDD4 KO cells (Fig 5B). The identities of secondary ubiquitin ligases for IFITM3 are still unknown. Of particular interest are the ubiquitin ligases capable of modifying the truncated Δ1–21 splice variant of human IFITM3, which was not significantly ubiquitinated by NEDD4 (Fig 2B). Identifying the ubiquitin ligases that modify this disease-associated variant may implicate crucial differences in the stability and degradative pathways potentially underlying the defect possessed by this protein. Nonetheless, our data clearly implicate NEDD4 as the primary E3 ubiquitin ligase for IFITM3, and demonstrate that NEDD4 is essential for maintaining low steady state IFITM3 levels.

This current work is in contrast to a prior study that concluded the IFITM3 PPxY motif was not involved in regulating the levels or antiviral activity of overexpressed IFITM3 [36]. However, this previous work did not directly assess ubiquitination of IFITM3 upon mutation of the PPxY motif. Additionally, our experiences studying IFITM3 ubiquitination here and in our prior work suggest that when examining overexpressed IFITM3 constructs, ubiquitination has only subtle effects on total protein levels detected by Western blotting despite significant effects on the IFITM3 half-life^13,20^. Thus, overexpression likely masked any effects of mutating the PPxY motif on the parameters previously tested [36].

Our study has uncovered a novel mechanism by which NEDD4 indirectly promotes cellular entry of influenza virus by decreasing IFITM3 levels (Figs 5, 6 and 7). Although this work is the first of its kind to identify NEDD4 as a negative regulator of IFITM3 levels, NEDD4 is well described to be necessary for the replication of several important RNA viruses. For example, NEDD4 interacts with proline-rich motifs in the viral late budding domains of Ebola virus [49], rabies virus [50], and HIV [51]. Mono-ubiquitination of these domains promotes efficient budding and viral egress necessary for productive viral spread. However, it remains to be determined whether inhibition of NEDD4 will serve as an effective *in vivo* antiviral strategy, particularly since NEDD4 is a developmentally essential molecule as demonstrated by the embryonic lethality of NEDD4 KO mice [52]. Likewise, NEDD4 has been implicated in regulation of insulin-like growth factor signaling [53], T-cell-mediated immunity [54,55], and tumor suppression [56]. On the other hand, neuron- and skeletal muscle-specific NEDD4 KO mice are viable [57,58], and NEDD4 is naturally inhibited by ISG15 during virus infections [29,30], perhaps suggesting that short-term inhibition of NEDD4 can occur without adverse effects. Additional experimentation will be needed to answer these vital questions, and this will be aided by the development of selective NEDD4 inhibitors, which is an area of active investigation [59,60]. Overall, our study identifies inhibition of NEDD4 as a novel strategy for preventing infection by influenza virus and other IFITM3-sensitive viruses through the increased accumulation of the antiviral restriction factor IFITM3.

## Materials and Methods

### Cell culture, transfections, and siRNA knockdowns

All cell lines used in these studies (HEK293T, A549, NCI-H358, NCI-H2009, and MEFs) were cultured in DMEM supplemented with 4.5 g/L D-glucose, L-glutamine, 110 mg/L sodium pyruvate, and 10% fetal bovine serum (Thermo Scientific) at 37°C and 5% CO_2_ in a humidified incubator. HEK293T and A549 cells were purchased from ATCC. NCI-H358 and NCI-H2009 cells were obtained from the ATCC and provided to us by Dr. Gustavo Leone (The Ohio State University). NEDD4 WT and KO MEFs used in this study were generated by Dr. Hiroshi Kawabe (Max Planck Institute)[38] and were kindly provided to us by Dr. Matthew Pratt (University of Southern California) who also generated the retrovirally reconstituted control cell lines. For Western blotting, cells were plated for 90% confluency in 6-well plates for 24 h prior to transfection with 2 μg/well of plasmids using Lipofectamine 2000 (Invitrogen). For microscopy, MEFs were plated for 50% confluency on glass coverslips in 12-well plates for 24 h prior to overnight treatment with IFN-α (BEI Resources). IFITM3 constructs were expressed from the pCMV-HA or pCMV-myc vectors (Clontech) as described previously [2,4,23]. IFITM3 mutants were made using the QuikChange Multi site-directed mutagenesis kit (Stratagene). Plasmids expressing HA-NEDD4 and HA-NEDD4-C867A were obtained from Addgene (plasmids 27002 and 26999, deposited by Dr. Joan Massagué, Memorial Sloan Kettering Cancer Center)[61], and plasmids expressing FLAG-NEDD4, FLAG-NEDD4-ΔWW, and HA-CBL-B were kindly provided by Dr. Jian Zhang (The Ohio State University). FLAG-SMURF1 and FLAG-SMURF2 were obtained from Addgene (plasmids 11752 and 11746, desposited by Jeff Wrana, University of Toronto)[62].

IFITM3 knockdown in MEFs was performed using Silencer Select Ifitm3 siRNA (Ambion, catalog no. 4390816) and negative control (Ambion, catalog no. 4390844). Human NEDD4 knockdown in A549, NCI H358, and NCI H2009 cells was performed using Dharmacon ON-TARGETplus SMARTpool Human NEDD4 (GE Healthcare, catalog no. L-007178-00) and Dharmacon ON-TARGETplus Control Pool Non-targeting control (GE Healthcare, catalog no. D-001810-10-20). siRNAs were transfected into cells using Lipofectamine RNAiMax transfection reagent (Invitrogen). Transfection of siRNA was performed for 24 h for mIFITM3 knockdown, and 48 h for NEDD4 knockdown. For Western blotting, cells were lysed with 1% Brij buffer (0.1 mM triethanolamine, 150 mM NaCl, 1% BrijO10 (Sigma), pH 7.4) containing EDTA-free protease inhibitor mixture (Roche) and 25 μM MG132 (Sigma). Immunoprecipitations were performed using EZview Red anti-c-myc or anti-HA affinity gel (Sigma), or with Protein G Plus Agarose Suspension (Calbiochem) in conjunction with anti-mIFITM3. Chloroquine, bafilomycin, and leupeptin were purchased from Sigma.

### Co-immunoprecipitation (Co-IP) assays

Co-immunoprecipitation assays were adapted from a previously described protocol [63]. HEK293T cells were co-transfected overnight with plasmids expressing myc-hIFITM3 and FLAG-NEDD4. Cells were washed twice with PBS, lysed on ice in Triton X-100 lysis buffer (50 mM Hepes, pH 7.5, 150 mN NaCl, 1% Triton X-100, 10% glycerol, 1.5 mM MgCl_2_, 1.0 mM EGTA, 10 μg/mL leupeptin, 10 μg/mL aprotinin, 10 μg/mL pepstatin, and 1 mM PMSF) for 5 min, and centrifuged at 1,000 x g for 5 min at 4°C. 50 μg of cell lysate was set aside for each sample in order to evaluate, via Western blotting, expression of myc-hIFITM3, FLAG-NEDD4, and GAPDH as a loading control. Equal concentrations of cell lysate were immunoprecipitated using 15 μL EZview Red anti-c-myc or anti-FLAG affinity gel (Sigma) per sample for 1 h at 4°C with gentle nutation. Immunoprecipitations were washed three times with lysis buffer and examined by Western blotting with both anti-myc and anti-FLAG for each immunoprecipitate.

### Western blotting and antibodies

Western blotting was performed with anti-myc (Developmental Studies Hybridoma Bank at the University of Iowa, deposited by Dr. J. Michael Bishop, catalog no. 9E 10), anti-HA (Clontech, catalog no. 631207), anti-hIFITM3 (Proteintech Group, catalog no. 11714-1-AP), anti-mIFITM3 (Abcam, catalog no. ab65183), anti-NEDD4 (Millipore, catalog no. 07–049), anti-FLAG (Sigma, catalog no. F7425), anti-actin (Abcam, catalog no. ab3280), or anti-GAPDH (Invitrogen, catalog no. 398600) antibodies. All primary antibodies were used at a 1:1000 dilution. Secondary antibodies, Goat Anti-Mouse IgG, HRP conjugate (Millipore catalog no. 12–349), Goat Anti-Rabbit IgG, HRP-linked (Cell Signaling, catalog no. 70745), and Goat Anti-Mouse, IgG1 Gamma 1 Heavy Chain Specific (SouthernBiotech, catalog no. 1070–05, specifically used for detecting immunoprecipitated protein ubiquitination) were all diluted at 1:20,000.

### *In vitro* ubiquitination

HEK293T cells were transfected overnight with plasmid expressing HA-hIFITM3. Protein collected from all wells of one 6-well plate was immunoprecipitated using anti-HA affinity gel and was washed extensively. Immunoprecipitated protein on affinity gel was resuspended in PBS. 10% of the retrieved protein was used in each reaction containing 500 μM ubiquitin or ubiquitin mutants (Boston Biochem, catalogue nos. U-100H, UM-K630, or UM-K480), 0.5 μM UbcH5b E2 ligase (Boston Biochem, catalogue no. E2-622), 100 nM UBE1 E1 ligase (Boston Biochem, catalogue no. E305), and 1x Ubiquitin Conjugation Reaction Buffer containing ATP (Boston Biochem, catalogue no SK-10) in the presence or absence of 100 ng recombinant human NEDD4 (Sigma, catalogue no. SRP0226). Reactions were allowed to proceed at 37°C for 1 h and were stopped by boiling for 5 min. The reactions were then diluted 1:100 in ice cold 1% Brij buffer, and IFITM3 was re-immunoprecipitated at 4°C using newly added anti-HA affinity gel prior to Western blot analysis.

### Fluorescence microscopy

Cells were fixed for 10 min with 3.7% paraformaldehyde, permeabilized with 0.1% Triton X-100 in PBS for 10 min, and blocked for 10 min with 2% FBS in PBS. Primary antibodies, anti-mIFITM3 (Fragilis, Abcam, catalogue no. ab15592) (1:500), anti-NEDD4 (1:500), and anti-LAMP1 (Santa Cruz Biotechnology, catalogue no. sc-19992), and Alexa Fluor-labeled anti-mouse and anti-rabbit secondary antibodies (Life Technologies, 1:1000) or anti-rat DyLight 550-labeled secondary antibody (Abcam, catalogue no. ab96888, 1:1000) were diluted in 0.1% Triton X-100 in PBS. Cells were treated with antibodies sequentially for 20 min at room temperature and washed five times with 0.1% Triton X-100 in PBS after each antibody treatment. Glass slides were mounted in ProLong Gold antifade reagent containing DAPI (Life Technologies). Images were captured using a Fluoview FV10i confocal microscope (Olympus).

### Infections and flow cytometry

Influenza viruses A/Puerto Rico/8/1934 (H1N1, PR8), a PR8 reassortant virus possessing the hemagglutinin and neuraminidase genes from A/Aichi/2/1968 (H3N2, X-31), A/Victoria/361/2011 (H3N2), and B/Texas/06/2011 were propagated in 10-day embryonated chicken eggs (purchased as day 0 eggs from Charles River Laboratories) for 48 h at 37°C as described previously [64]. PR8 and X-31 were provided to us by Drs. Bruno Moltedo and Thomas Moran (Mount Sinai School of Medicine) and the 2011 virus isolates were obtained from BEI Resources sponsored by the NIH/NIAID. SeV expressing green fluorescent protein (SeV-GFP)[65] was generated by Dr. Dominique Garcin (University de Geneve) and provided to us by Dr. Mark Peeples (Nationwide Children’s Hospital Research Institute). SeV-GFP was propagated in 10-day embryonated chicken eggs for 40 h at 37°C as described previously [66]. VSV G-pseudotyped retrovirus expressing green fluorescent protein was generated by transfection of the viral vector pLenti-CMV-GFP-puro (Addgene plasmid 17448, deposited by Dr. Eric Campeau)[67] and packaging plasmids (provided by Dr. Li Wu, The Ohio State University) along with plasmid expressing VSV G into HEK293T cells. Direct inhibition of GFP production by IFITM3 was not expected since this is driven by the CMV immediate early promoter and bypasses retrovirus-specific expression machinery [68]. Media was changed 18 h post-transfection, and media containing virus was then harvested 48 h post-transfection. Virus-containing media was centrifuged at 1200 x g for 5 min, filtered with 0.45 μm filters, frozen, stored at -80°C, and used for infection at a dose that provided approximately 70% infection of WT MEFs. MEFs were infected with IAV PR8, X-31, and H3N2 2011 strains, SeV and IBV at a multiplicity of infection of 5.0 or 10.0. MEFs were infected for 24 h, except in the case of VSV G-pseudotyped retrovirus infections, which were analyzed after 48 h of infection. A549 cells were infected with IAV strain PR8 at a multiplicity of infection of 2.5 for 6 h. Infected cells were washed with PBS and harvested in 0.25% trypsin EDTA. Cells were fixed in 3.7% paraformaldehyde for 10 min and permeabilized with 0.1% Triton X-100 for 10 min. IAV infected cells were stained with anti-influenza nucleoprotein (Abcam, catalog no. ab20343, 1:333) directly conjugated to Alexa Fluor 647 using a 100 μg antibody labeling kit (Life Technologies). IBV infected cells were stained with anti-IBV nucleoprotein (Thermo Scientific catalogue no. MA1-80712, 1:1000) followed by anti-mouse secondary antibodies conjugated directly to Alexa Fluor 488 (Life Technologies). Measurement of SeV and VSV G-pseudotyped retrovirus infection rates was done by detecting virus-encoded GFP. All antibodies were diluted in 0.1% Triton X-100 in PBS, and cells were stained for 20 min. Cells were washed three times with 0.1% Triton X-100 in PBS after each antibody treatment. PBS was used for final resuspension of cells for flow cytometric analysis using a FACSCanto II flow cytometer (BD Biosciences). Results were analyzed using FlowJo software.
